# Infra-slow (<0.1 Hz) Modulation of Human Brain Pulsations in Awake and Sleep States

**DOI:** 10.1523/JNEUROSCI.2282-25.2026

**Published:** 2026-06-05

**Authors:** Tommi Väyrynen, Heta Helakari, Vesa Korhonen, Johanna Tuunanen, Niko Huotari, Janne Kananen, Seyed-Mohsen Ebrahimi, Ahmed Elabasy, Matti Järvelä, Katariina Hautamäki, Katariina Laurén, Lauri Raitamaa, Ulla Salmi, Johanna Piispala, Mika Kallio, Vesa Kiviniemi

**Affiliations:** ^1^Oulu Functional Neuroimaging, Faculty of Medicine, University of Oulu, Oulu 90014, Finland; ^2^Research Unit of Health Sciences and Technology, Faculty of Medicine, University of Oulu, Oulu 90014, Finland; ^3^Medical Research Center Oulu, Oulu University Hospital and University of Oulu, Oulu 90220, Finland; ^4^Radiology, Diagnostics, Oulu University Hospital, Oulu 90220, Finland; ^5^Clinical Neurophysiology, Diagnostics, Oulu University Hospital, Oulu 90220, Finland; ^6^Biocenter Oulu, Faculty of Biochemistry and Molecular Medicine, University of Oulu, Oulu 90220, Finland

**Keywords:** brain pulsations, cross-frequency coupling, CSF dynamics, fMRI, neurofluid, sleep

## Abstract

The human brain exhibits three propagating pulsations, namely, cardiovascular, respiratory, and infra-slow fluctuations (ISFs <0.1 Hz), which are thought to contribute to the flow of intracranial fluids. While their pulsation characteristics have been extensively studied, their mutual dependencies have not been systematically investigated. Using ultrafast whole-brain magnetic resonance encephalography fMRI sequence, we analyzed the frequency domain up to 5 Hz for cross-frequency oscillatory interactions in awake and NREM sleep states of 23 (13 F, 10 M) healthy volunteers. Using phase transfer entropy analysis, we found that in the awake state the resting-state network (RSN) activity largely predicted the neurofluid (NF) signal changes. NREM sleep was associated with increased power of ISF and with altered directed coupling patterns between RSN and NF components. Importantly, within these independent signal sources, we found three distinct cross-frequency coupling frequency ranges occurring at ISF (<0.1 Hz), respiratory (∼0.25 Hz), and cardiovascular (∼1 Hz) frequencies, where the slower pulsations generally predicted the faster ones, except for a finding of inverted cardiorespiratory coupling in NREM-2 sleep. These results suggest the presence of directional ISF mediated mechanisms underlying brain pulsations that contribute to driving the intracranial fluid transfer processes.

## Significance Statement

Cerebrospinal fluid (CSF) flow is essential for brain fluid homeostasis and interstitial metabolite clearance. The human brain exhibits three types of intracranial pulsations linked to CSF flow, which are particularly distinct during sleep, when fluid clearance processes are most active. We hypothesized that these pulsations, despite their independent sources, interact with each other to facilitate CSF flow. Using fMRI during wakefulness and non-rapid eye movement sleep, we investigated cross-frequency coupling patterns up to 5 Hz within the brain. The results revealed novel cross-frequency coupling bands in the human brain, in which infra-slow fluctuation dynamics predicted faster brain pulsations, potentially contributing to increased perivascular clearance during sleep.

## Introduction

The human brain relies on circulation of cerebrospinal fluid (CSF) through low resistance perivascular spaces, allowing the influx of CSF into brain cortex and mixing with the interstitial fluid (Iliff et al., 2012). This transport allows removal of metabolic waste products, which is essential for normal brain function (Kelley and Thomas, 2023). Sleep-associated increase of the interstitial space between neurons and astrocytes promotes more efficient CSF flow (Xie et al., 2013). Conversely, decreased sleep duration has been linked to accumulation of amyloid-β (Shokri-Kojori et al., 2018).

Considerable effort has been put into understanding the physiological drivers of net CSF flow in brain parenchyma. The three types of intracranial pressure fluctuations, infra-slow fluctuations (ISFs), respiratory, and cardiovascular pulsations, oscillate within the human brain and can be detected with rapidly sampled fMRI magnetic resonance encephalography (MREG) sequence. All three pulsations have been suggested to contribute to net CSF flow by mechanically displacing perivascular CSF (Hadaczek et al., 2006; Iliff et al., 2013; Dreha-Kulaczewski et al., 2015, 2017; Mestre et al., 2018; van Veluw et al., 2020; Bojarskaite et al., 2023; Hauglund et al., 2025).

Initial studies suggested that cardiovascular pulsatility drives the inflow of subarachnoid CSF to the cerebral cortex of mice (Hadaczek et al., 2006; Iliff et al., 2013; Mestre et al., 2018). Other research lines suggested that downward flow of venous blood during inspiratory phase of respiration is counterbalanced by upward flow of CSF toward head (Dreha-Kulaczewski et al., 2015, 2017; Kiviniemi et al., 2016).

Most recently, arterial vessel wall fluctuations, known as vasomotor waves, have been proposed to propel brain CSF flow (Hauglund et al., 2025). Optogenetic stimulation of noradrenergic neurons in the locus ceruleus evoked anticorrelated vasomotor and CSF fluctuations in mice (Hauglund et al., 2025). By current understanding, the controlled release of the vasoconstrictor norepinephrine (NE) from ascending projections of the locus ceruleus coordinates widespread vasomotor waves during sleep (Osorio-Forero et al., 2021; Kjaerby et al., 2022; Lüthi and Nedergaard, 2025).

ISFs in resting-state fMRI arise from multiple interacting physiological processes, including spontaneous neuronal activity, slow vasomotor fluctuations of arterial vessel walls, as well as other systemic processes (Drew, 2019). In humans, blood volume fluctuations are also accompanied by opposing changes in cortical CSF volume during non-rapid eye movement (NREM) sleep stages 1−2 (Borchardt et al., 2021; Väyrynen et al., 2026). Moreover, the signal power and propagation speed of these ISFs increase during NREM sleep (Helakari et al., 2022; Elabasy et al., 2026) and further synchronize the arterial vascular changes with concomitant venous BOLD signal (Tuunanen et al., 2024). Disruption of the characteristics of brain pulsations occur in several neurological conditions. For example, patients with type 1 narcolepsy show increased ISFs but reduced cardiovascular pulsatility in awake state compared with healthy controls (Järvelä et al., 2025). Altered pulsation dynamics have been also reported in several central nervous system disorders (Kananen et al., 2020, 2022; Tuovinen et al., 2020; Rajna et al., 2021; Poltojainen et al., 2022).

These findings raise the possibility that the three physiological pulsations are comodulated. The limited temporal resolution of conventional echoplanar imaging BOLD sequences constrains the detectable frequency range, making it difficult to capture fast and slow oscillatory components simultaneously (Huotari et al., 2019). As a result, there has been no systematic investigation of the hemodynamic coupling patterns in the human brain.

To address this issue, we used a T2*-based fMRI MREG sequence sampled at 10 Hz to accurately capture pulsation dynamics. We searched for cross-frequency coupling patterns using phase–amplitude coupling estimator (PAC; Canolty and Knight, 2010) during EEG-verified awake and NREM1-2 sleep in healthy volunteers. We further employed phase transfer entropy (TE) to estimate the information transfer between the coupled frequency ranges (Lobier et al., 2014), testing our hypothesis that ISFs predict faster cardiorespiratory amplitudes.

## Materials and Methods

### Study design and measurements

Our study group consisted of *N* = 23 healthy controls (13 females, 10 males, mean age 27.0 years), who had been investigated in our prior studies (Helakari et al., 2022, 2023). The volunteers participated in two separate fMRI scanning sessions, each lasting ∼1 h: one scan measured wakefulness and the other aimed to capture sleep episodes. Approximately half (*N* = 13) of the subjects had undergone one night of sleep deprivation prior to scanning to facilitate rebound sleep. Their sleep deprivation had been verified by recordings obtained the prior night using smart rings (Oura Health Oy). Written informed consent had been obtained from all participants in accordance with the Declaration of Helsinki. Our study was screened and approved by the Pohde Regional Ethics Committee of the Northern Ostrobothnia Hospital District. In synchrony with the fMRI, we obtained EEG recordings to delineate awake epochs from sleep states and to separate NREM-1 and NREM-2 sleep stages. Sleep stage classification was performed manually in 30 s EEG epochs by experienced neurophysiologists (J.P., M.K.) according to the criteria defined by American Academy of Sleep Medicine (AASM).

We used a 3 T Siemens MAGNETOM Skyra scanner with 32-channel head coil to capture the structural and functional MREG image series. The imaging parameters for structural T1-weighted 3D MPRAGE scans were as follows: TR, 1,900 ms; TE, 2.49 ms; FA, 9°; FOV, 240 mm; and slice thickness of 0.9 mm. An MREG sequence was used to measure functional datasets using the following parameters: TR, 100 ms; TE, 36 ms; FA, 5°; FOV, 192 mm. The MREG sequence undersamples *k*-space, reaching a 10 Hz sampling frequency with voxel size of 3 mm (Assländer et al., 2013). The crusher gradient of 0.1 was optimized for physiological signals sources, which also mitigates slow drifts and stimulated echoes. The image reconstruction used L2-Tikhonov regularization with a lambda value of 0.1, as determined by the L-curve method (Hugger et al., 2011). To reduce B0-field artifacts, we used dynamic off-resonance correction in *k*-space.

EEG was recorded using the GES 400 system (Magstim EGI), consisting of a direct current-coupled amplifier (Net Amps 400), and 256-electrode MRI compatible electrode net (HydroCel Geodesic Sensor MR Net). We used electrode “Cz” located at the vertex of the head as the reference electrode. The EEG recordings were conducted with sampling rate of 1 kHz, except for three sleep and five awake scans, where 250 Hz sampling was used erroneously.

### Data preprocessing steps

**Figure 1.**
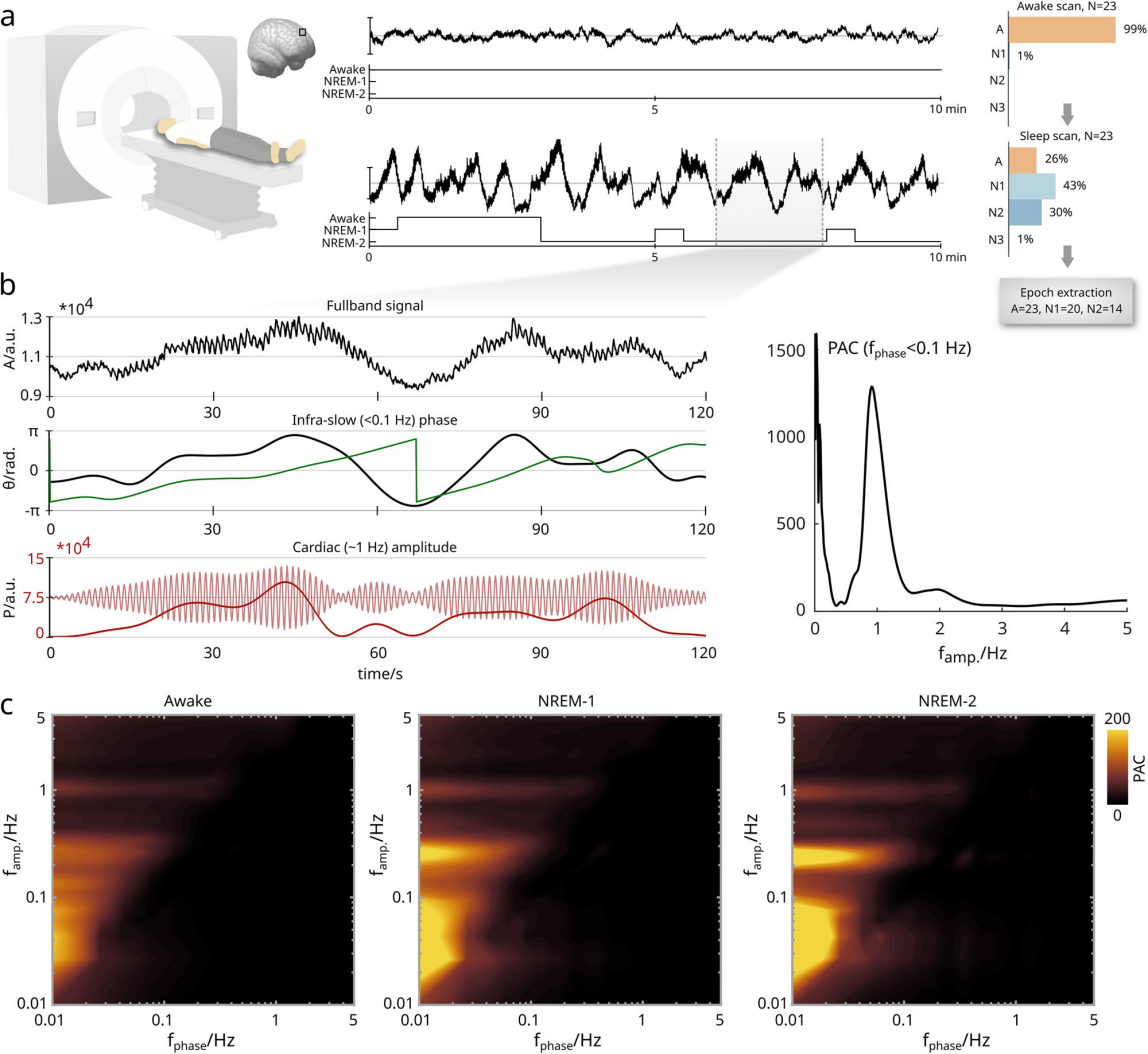
Infra-slow fluctuation (ISF, <0.1 Hz) changes are coupled with known physiological pulsation powers. ***a***, Example signal for an entire 10 min sequence length during awake and sleep sessions, along with EEG-derived hypnograms. On the right, the bar plot depicts the mean proportion of wakefulness and sleep stages captured during the scans relative to the total scan length. ***b***, Top row illustrates a 2 min full bandwidth signal during NREM-2 sleep, where phase–amplitude coupling (PAC) of ISF phase and cardiac amplitudes (∼1 Hz) is visible. The middle row shows the ISF filtered signal and the corresponding phase time series (green). The bottom row shows the cardiac frequency filtered signal and associated cardiac power fluctuations over time (red). PAC of the example signal is shown next to signals, which demonstrates that ISF phase couples with cardiac power ∼1 Hz. ***c***, Average group level PAC estimates across frequency space in awake, and NREM-1 and NREM-2 states, reveal three coupling frequency ranges, which increase in magnitude as a function of sleep stage. The *x*-axis illustrates the phase frequency, whereas the *y*-axis indicates the frequency of amplitude, and color scale represents the PAC coupling magnitude.

MREG image processing was performed using the FSL library (Jenkinson et al., 2012). We used a cutoff frequency of 0.008 Hz to high-pass filter the datasets and then applied MCFLIRT motion correction and further despiking to eliminate artifactual spikes (Cox, 1996). The structural T1 images were used to spatially normalize functional images into the MNI152 standard space. Two-minute length segments of wakefulness and NREM1-2 sleep were identified for each participant using the EEG-based sleep scores and utilized in further analysis. The NREM-1 dataset consisted of *N* = 20 (10 females, 10 males, average age, 26.2 years), and the NREM-2 set consisted of *N* = 14 (7 females, 7 males, average age, 27.8 years; Fig. 1*a*).

Ballistocardiographic and gradient switching related EEG artifacts were removed using template subtraction (Allen et al., 1998, 2000) in Brain Vision Analyzer (Brain Vision Analyzer v.2.1, Brain Products).

### Analysis of independent components

**Figure 2.**
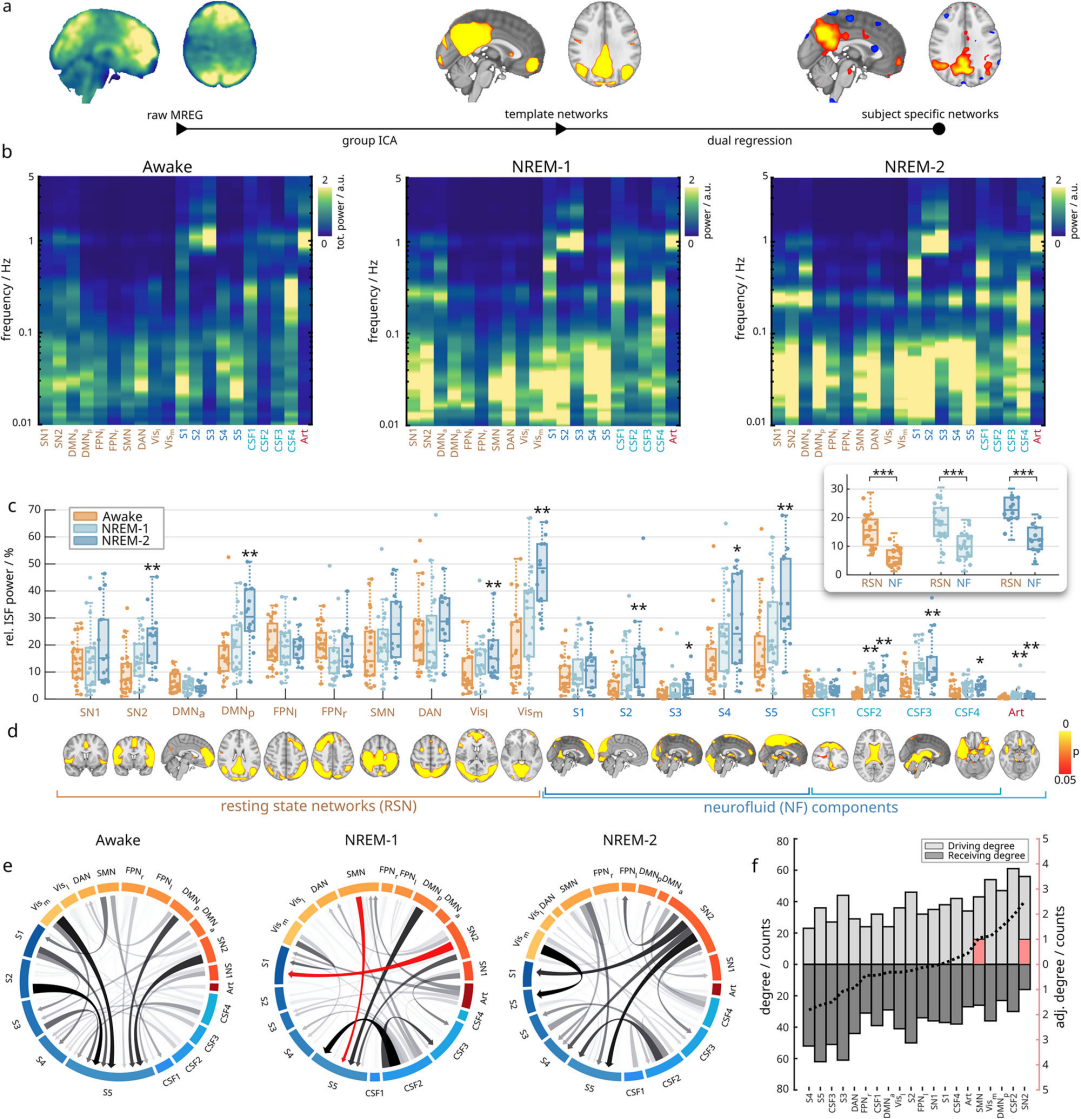
Spectral characteristics of resting-state networks (RSNs) and neurofluid (NF) components of brain. ***a***, Independent component analysis (ICA) was used to separate RSN from spatially independent NF sources. ***b***, Average component spectra are depicted for the 0.01–5 Hz frequency range in awake and NREM-1/NREM-2 sleep states. ***c***, Infra-slow (ISF <0.1 Hz) powers relative to total signal power levels, with units in percentiles. Relative ISF power within ICs were compared between awake and sleep states. Average RSNs and NF component powers were also compared with each other in the additional axis. False discovery rate (FDR) adjusted statistical significance is denoted by asterisks (*p*_adj_ < 0.05*, 0.01**, 0.001***). ***d***, Spatial maps of ICA template networks in the most representative horizontal, coronal, or sagittal slice, with assignment of visually labeled components to classical RSN (left) and NF (right) components. ***e***, Surrogate-normalized phase transfer entropy (TE) prediction patterns between ICs are visualized as network diagrams. Significant connections after FDR correction are highlighted in red color. Arrows indicate the direction of the interactions. ***f***, Degree of coupling across ICs combined from all arousal states. The left ordinate axis shows the number of surrogate-adjusted significant connections, while the right axis shows the FDR-adjusted degree.

Group ICA was calculated with the FSL MELODIC package (Beckmann et al., 2009) using a temporal concatenation approach and including the full-length scans across awake and sleep recordings (Fig. 2*a*,*d*). To circumvent the tendency for components to split into subcomponents in higher model orders (Abou-Elseoud et al., 2010), we reduced the data dimensionality to 20 components using PCA and used the variance normalization option to ensure equal weighting across voxels.

Subsequently, a neuroradiologist (VK) visually inspected and labeled the template networks (Kiviniemi et al., 2003, 2009) into components representing functionally connected RSNs and NF-related sources. RSNs reflect synchronous fluctuations in brain activity and appear as spatially independent signal sources in MREG data (Beckmann et al., 2009; Kiviniemi et al., 2009). In contrast, NF components are dominated by physiological pulsations and are anatomically associated with CSF spaces and major blood vessels.

Ten canonical RSNs were identified: anterior and posterior divisions of the default mode network (DMN_a_, DMN_p_), associated with internally directed cognitive processes; two salience network components (SN1, SN2), involved in stimuli importance detection and network switching; left and right frontoparietal networks (FPN_l_ and FPN_r_), supporting cognitive control and working memory; the dorsal attention network (DAN), regulating voluntary attention; the sensorimotor network (SMN); and visual networks located in medial (Vis_m_) and lateral (Vis_l_) visual cortices.

Moreover, 10 ICs were classified as NF components. One component represented the major cerebral arteries (Art), and five components (S1–S5) corresponded to different segments of the superior sagittal sinus, showing pulsatile activity partly coupled with central CSF spaces due to venous outflow dynamics (Fig. S1). The remaining four components were associated with CSF circulation pathways: frontal CSF spaces (CSF1), the lateral ventricles (CSF2), the third ventricle, aqueduct, infratentorial CSF and the fourth ventricle (CSF3), and lastly the temporal Sylvian fissure and midline CSF spaces (CSF4).

Subject-specific networks were obtained through FSL's dual regression method (Beckmann et al., 2009). This procedure involved fitting template networks to the subject-specific fMRI datasets, resulting in a set of subject-specific independent components corresponding to the templates.

We calculated spectral powers extending from 0.01 to 5 Hz (Fig. 2*b*,*c*) using a wavelet convolution approach (Cohen, 2014) and then obtained instantaneous power estimates by squaring the complex magnitude results $P=|z{|}^{2}$. We further estimated the ISF band power (0-01-0.1 Hz) using integral approximation and rectangular windowing. Instead of focusing exclusively on absolute ISF powers, we obtained relative power levels by dividing the ISF powers by total signal power to avoid effects of altered overall signal power levels.

### Estimating frequency-specific coupling ranges

To quantify cross-frequency coupling across the whole frequency range, we used PAC, which quantifies the relationship between the phase of one frequency and power in another (Canolty and Knight, 2010; Cohen, 2014). We calculated PAC with wavelet frequencies across the 0.01 to 5 Hz range using 300 linear increments. Wavelet convolution with complex Morlet wavelets (*N* = 6) was used to filter the signals at each frequency step. From the convolution results, we further extracted the phase and power corresponding to each frequency bin. PAC was defined as $\left|n^{-1}\sum_{t=1}^{n}a_{t}e^{i\phi_{t}}\right|$, where a is the power at time point t, i is the imaginary operator, ϕ is phase angle in radians, and n is the number of time points (Cohen, 2014). We iterated PAC calculations for each (*f*_phase_, *f*_power_) frequency combination, producing two-dimensional coupling estimates. A random sampling approach (*N* = 3,405) without replacement was used to assess the whole-brain PAC, utilizing 5% voxels of the whole-brain volume (Fig. 1*c*).

**Figure 3.**
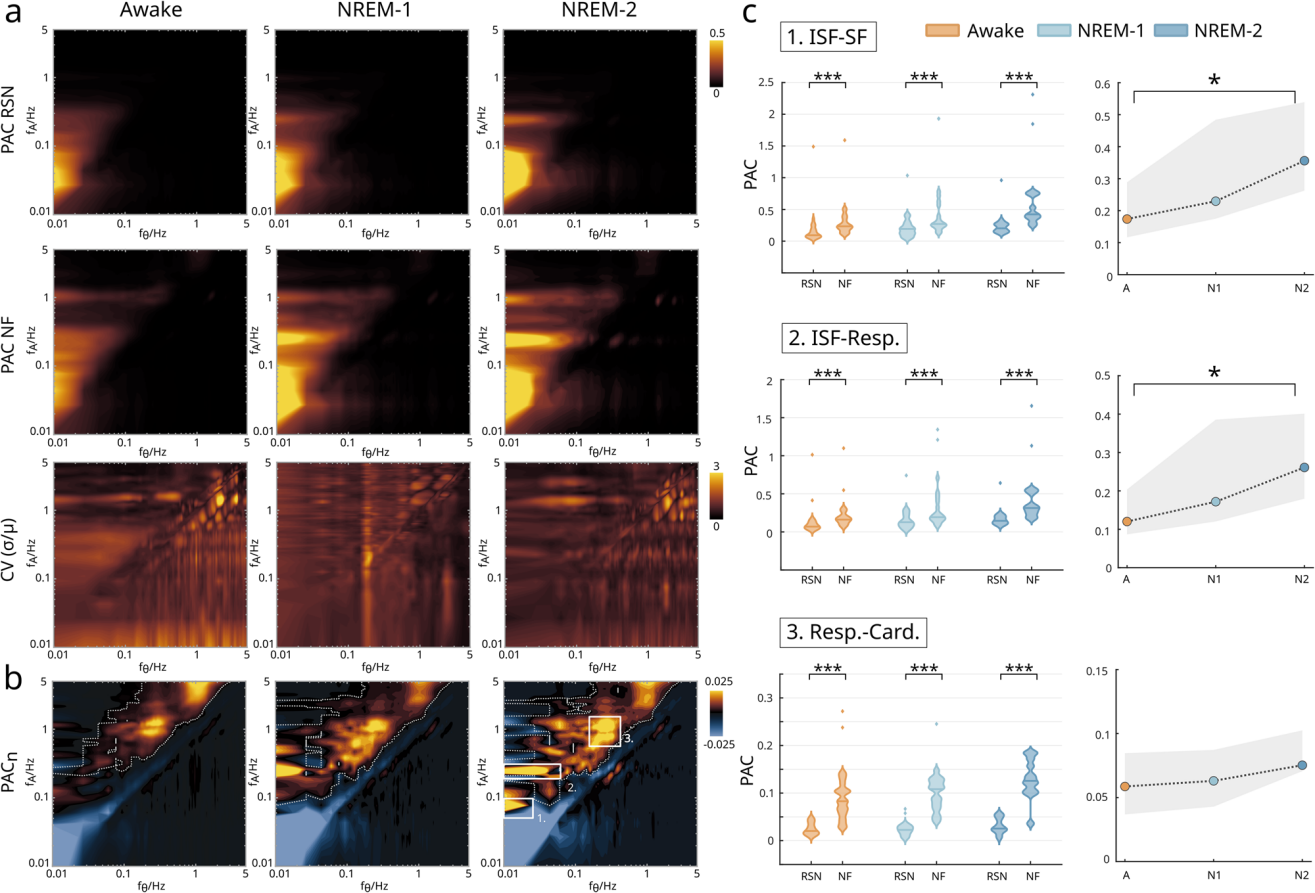
Cross-frequency coupling ranges associated with resting-state networks (RSNs) and neurofluid (NF) components. ***a***, The frequency–frequency maps of unthresholded phase–amplitude coupling (PAC) between phase (*x*-axis) and amplitude (*y*-axis) frequencies across RSN and NF components in awake and sleep stages. Intersubject variability is presented as coefficient of variation (CV) maps for the average PAC maps, where higher values indicate greater variability relative to the mean PAC. ***b***, Surrogate mean subtracted PAC_n_ maps of combined independent components (ICs) illustrate PAC values significantly higher from background noise (dotted line, cluster analysis, *p* < 0.05*). ***c***, Raw PAC values extracted for coupling ranges (as numbered 1–3) averaged over RSN and NF. Panel ***c***, right tiles show the grand average PAC over for wakefulness and sleep states, indicating increasing coupling with sleep stage. Statistical significance is denoted by asterisks (*p*_adj_ < 0.05*, 0.01**, 0.001***).

PAC patterns were further assessed across the frequency space with ICs (Fig. 3). Having obtained reduced data dimensionality, we utilized surrogate data (Ns = 100) to evaluate the statistical significance of each *f*_phase_, *f*_power_ point. This approach revealed PAC high-coupling regions, which we analyzed in more detail. For each subject, we extracted their individual cardiac and respiration frequencies and static ISF and SF bands, to study their several coupling patterns (ISF–SF, ISF–Resp, and Resp–Card) in greater detail.

### Assessing directional dependencies

To calculate information transfer in the ranges with coupling, we used the phase TE approach (Lobier et al., 2014). This process involved first estimating the analysis lag, before proceeding to the actual TE estimation. Here, TE was estimated between the phase of the slow signal and the phase of the amplitude envelope of the fast signal (Vanhatalo et al., 2004; Palva et al., 2005; Väyrynen et al., 2023).

As in PAC magnitude analysis, we used static frequency bands for ISF and slow frequency bands. For the respiratory and cardiac bands, we used the individual peak cardiorespiratory frequencies as center frequencies for finite impulse response (FIR) bandpass filters, using a filter order of *N* = 10^3^ and band width of 0.2 Hz for respiratory and 0.1 Hz for cardiac frequency bands. Application of the mirroring technique with recursive filtering ensured accurate phase estimates.

**Figure 4.**
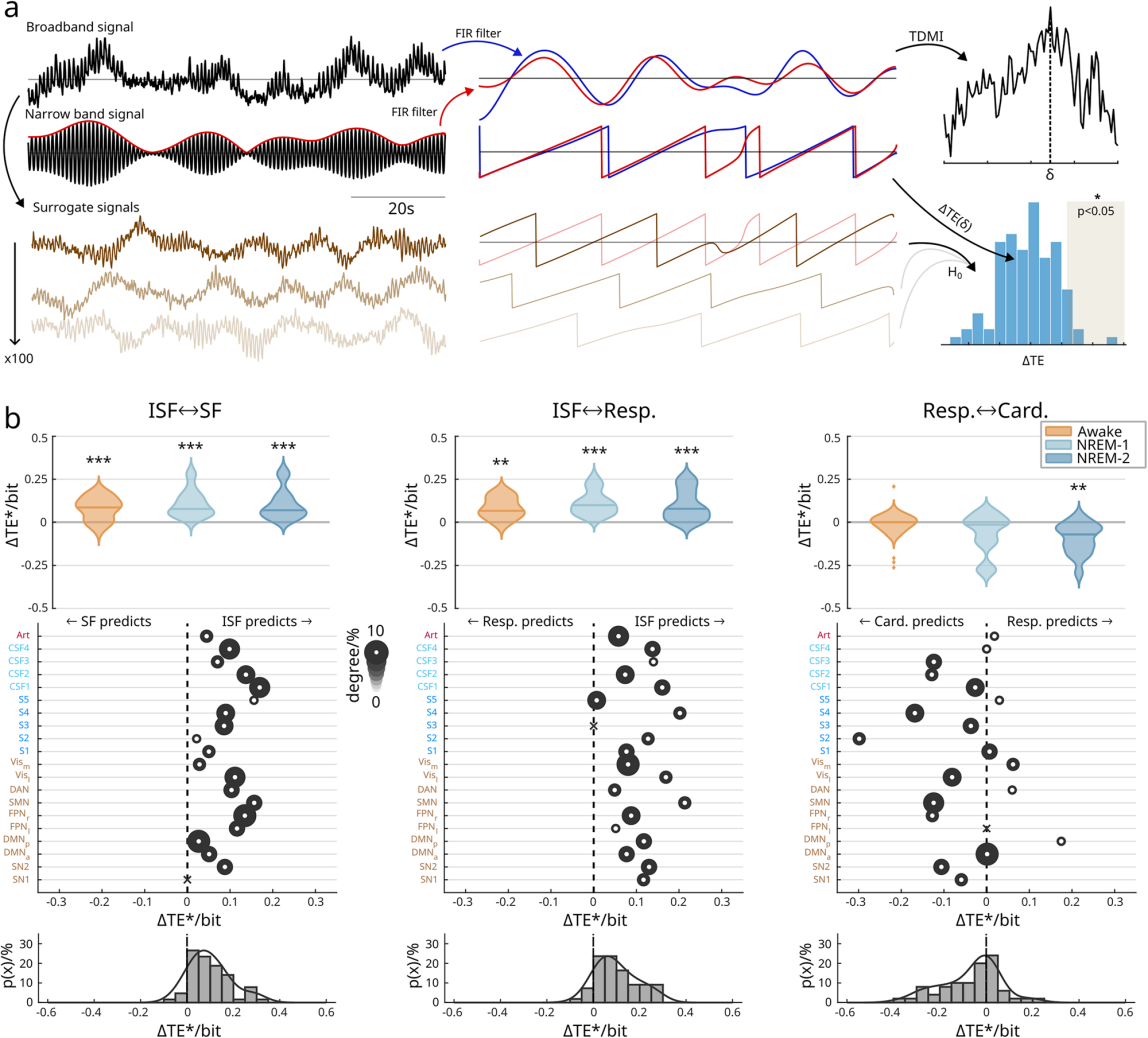
Infra-slow (ISF) network dynamics predict slow and respiratory frequency oscillations. ***a***, In the procedure for directional inferences, we first filtered the broadband signal into two target narrowband frequencies. The phase was extracted from the lower-frequency component, while the analytic envelope of the higher-frequency oscillations was computed and subsequently filtered at the corresponding lower frequency. Phase time series were then derived, and the optimal analysis lag was determined using time-delayed mutual information (TDMI). This lag was subsequently applied in estimating directional interactions via phase transfer entropy (TE). Statistical significance was assessed using surrogate data distribution generated from the broadband signal by the time-shift method. ***b***, Violin plot illustrates significant (*p* < 0.05) ΔTE between 1, ISF–SF; 2, ISF–Resp; and 3, Resp–Card coupling patterns in awake and NREM-1 and NREM-2 sleep states over all ICs. Below, the scatterplot depicts the contribution of each independent component (IC) as an average over subjects. The size of the circle represents the number of significant values in percentiles. At the bottom, probability density estimate collapsed over the IC, demonstrating that on average the ISF predicts SF and Resp fluctuations, while in Resp–Card coupling, cardiac pulsation is actually the net predictor of respiratory oscillations. Statistical significance is denoted by asterisks (*p*_adj_ < 0.05*, 0.01**, 0.001***).

We first estimated the shared information between the two pairs of variables using time-delayed mutual information (TDMI) analysis. In this approach, we assessed lags ranging from −2 to 2 s in 0.1 s step size. The lag value maximizing mutual information was used in the estimation of phase TE (Lobier et al., 2014), which proceeded by applying the Hilbert transform to acquire the analytical signals, using the bandpass filtered signals. For the slow signal, phase was extracted from the analytical signal. For the fast signal, we first calculated the analytical envelope, which we then filtered to the corresponding slow frequency band, followed by extraction of phase (Fig. 4*a*).

We then proceeded to calculate TE between the pair of phase time series with the TDMI analysis lag estimates (Fig. 4*b*). Here, we used a discrete estimator, essentially binning the data to represent the state space. Phase TE was then calculated within each IC and in the three identified coupling ranges in both interaction directions. Pairwise TE was then formulated into its directional form, defined as a difference ΔTE *=* TE(*x → y*)* − *TE(*y → x*).

The same approach was taken to estimate the connectivity between the RSNs and NF components (Fig. 2*e*). Here, we utilized the ISF filtered subject-specific networks to focus specifically on the lower end of the spectrum. As before, we likewise began by estimating the analysis lag that maximized mutual information between all combinations of the ICs. To this end, we used TDMI with possible lags ranging from ±5 s and then proceeded to calculate TE between all IC combinations using the optimal analysis lag.

### Statistical analysis

We did not make a priori calculation of sample size based on statistical methods. For all statistical tests in this exploratory study, we used an alpha level of 0.05 to infer statistical significance. The differences in relative ISF powers (Fig. 2*c*) were compared between the awake-NREM-1 and awake-NREM-2 contrasts using Wilcoxon rank-sum test, with the null hypothesis that the median ISF powers are equal between the arousal states. ISF power was also compared between RSN and NF components within each arousal state using Wilcoxon signed rank test. For both, we used Benjamini–Hochberg FDR correction to control false-positive results (Benjamini and Hochberg, 1995). A similar approach was taken to test the raw PAC values of IC components (Fig. 3*b*). We used the sign test and FDR correction to assess if the information transfer has a net directionality in the three observed coupling ranges (Fig. 4*b*).

Moreover, we utilized surrogate statistics (Theiler et al., 1992; Lachaux et al., 1999; Lobier et al., 2014) to perform model-free, empirical statistical analysis (Figs. 2*e*, 3*a*, 4). The null hypothesis distributions were built using Ns = 100 surrogates, where each surrogate signal was constructed using the time-shift method. In this approach, the original signal is split and reattached from a random time point, which preserves the signal autocorrelation structure of the original data, but removes any linear correlations.

Statistical significance of PAC frequency–frequency maps was further assessed using a nonparametric cluster-based permutation test controlling the familywise error rate, in which cluster-mass statistics from observed data were compared with a null distribution (*N* = 1,000) generated by sign-flipping technique, with significance set at *p* < 0.05 (Maris and Oostenveld, 2007; Winkler et al., 2014).

## Results

### Infra-slow (<0.1 Hz) phase is coupled with higher-frequency amplitudes in the human brain

To establish the frequency-specific coupling among pulsations in the human brain, we quantified PAC in the fMRI MREG signal across the frequency domain extending from 0.01 to 5 Hz (Fig. 1*a*,*b*). Voxel level analysis revealed three separate high-coupling regions involving ISF, respiratory, and cardiac frequencies, where coupling magnitudes seemed to increase progressively as a function of sleep stage, according to averages (Fig. 1*c*). The first coupled frequency range involved ISF amplitudes, which were coupled with even slower ISF phase changes: *f*_amp_ (0.02–0.1 Hz); *f*_phase_ (0.01–0.02 Hz). The second coupling range matched respiratory amplitudes, which were similarly coupled with ISF phase changes: *f*_amp_ (∼0.25 Hz); *f*_phase_ (0.01–0.05 Hz). The third coupling frequency range highlighted cardiac amplitudes, which were coupled with a wider phase bandwidth: *f*_amp_ (∼1 Hz); *f*_phase_ (0.01–0.1 Hz).

The coupling, especially of respiratory and ISF amplitudes, demonstrated higher mean PAC levels in comparison with cardiac amplitudes. NREM-1 and NREM-2 sleep stages were both associated with elevated average PAC values in contrast to awake state. Confirming our initial hypothesis, we found multiple coupled frequency bands in the hemodynamic signal, which corresponded to the well-known brain pulsation frequency ranges: ISF, respiratory, and cardiac range.

### Sleep enhances ISFs that are more prominent in resting-state networks than in neurofluid compartments

It has been unknown how the three brain pulsations are expressed in independent functional units of the human brain. To quantify power contributions of simultaneous cardiorespiratory and ISF vascular changes on neurofluid (NF) and on functionally connected brain networks, we computed the spatially independent components (IC). The ICs were categorized into NF components and classical resting-state networks (RSNs), after visual inspection by an expert neuroradiologist (V.Ki.; Fig. 2*a*,*d*). NF components were defined as spatially independent components that did not correspond to canonical RSNs and spatially colocalized with anatomical fluid containing structures, including CSF spaces and vascular structures. See also Figure S1 and Materials and Methods for a complete description of the ICs. We then assessed the component frequency domain by analyzing signal power levels up to 5 Hz in awake and sleep states.

As expected, fast cardiovascular pulsations were prominent in the IC labeled as the cerebral artery Art but were also strongly expressed in venous sinus S2 and S3 components (Fig. 2*b*). In contrast to NF components, RSNs in general exhibited less cardiovascular activity, except for the anterior default mode network DMN_a_ and salience network SN2 located close to the anterior and medial cerebral artery branches. Slower respiratory fluctuations were most dominant within CSF spaces CSF1, 3, and 4, but also visible in the venous S1 and S4 components, and in the lateral visual Vis_l_ and SN2 networks. The ISF powers were highly pronounced especially in venous S1, S4, and S5 and with RSNs in the dorsal attentional network DAN, DMN_p,a_, and SN2. As indicated by the average spectral powers, the ISF power was distributed across a broad frequency range below 0.1 Hz. The ICs showed unique ISF characteristics, with a patchy appearance rather than having a single frequency centered band.

We then proceeded to make a statistical assessment of the ISF powers relative to total signal power levels in awake and NREM1-2 sleep states (Fig. 2*c*, Table S1). Based on our previous voxel level studies (Helakari et al., 2022; Väyrynen et al., 2026), we hypothesized that ISF power would increase in the collective ICs during NREM sleep. The overall levels of ISF band powers were higher in RSNs (*P*_A_ = 15.7%, IQR = 8.8%; *P*_N1_ = 17.6%, IQR = 9.9%; *P*_N2_ = 22.7%, IQR = 7.2%) in comparison with NF-labeled components (*P*_A_ = 5.9%, IQR = 4.9%; *P*_N1_ = 9.9%, IQR = 7.2%; *P*_N2_ = 12.3%, IQR = 7.6%) in all arousal states *A*_RSN_-*A*_NF_: *Z* = 4.2, *p*_adj_ < 0.001***, N1_RSN_-N1_NF_: *Z* = 4.0, *p*_adj_ < 0.001***, N2_RSN_-N2_NF_: *Z* = 3.2, *p*_adj_ < 0.001*** (Fig. 2*c*). Eight of the 10 NF components showed significantly increased ISF power during sleep. In particular, the arterial component showed increased ISF band power in comparison with both NREM states (Art: A-N1: *Z* = −3.7, *p*_adj_ < 0.01**, A-N2: *Z* = −3.2, *p*_adj_ < 0.01**), even though absolute levels of ISF powers remained low and dominated by cardiac activity. All CSF components except for the frontal CSF1 component had increased power during sleep (CSF2: A-N1: *Z* = −3.8, *p*_adj_ < 0.01**, A-N2: *Z* = −3.5, *p*_adj_ < 0.01**). Similarly, all venous sinus components except for frontal S1 also showed increased power in the ISF range (S5: A-N1: *Z* = −1.7, *p*_adj_ = 0.1, A-N2: *Z* = −3.1, *p*_adj_ < 0.01**).

Only four among the ten classical RSNs studied showed significant increases in ISF band power during sleep (Fig. 2*c*). Both visual networks: lateral and medial increased ISF power (Vis*_m_*: A-N1: *Z* = −2.0, *p*_adj_ = 0.08, A-N2: *Z* = −4.2, *p*_adj_ < 0.01**). Also, posterior parts of DMN showed increased ISF power in NREM-2 sleep (DMN*_p_*: A-N1: *Z* = −0.9, *p*_adj_=0.5, A-N2: *Z* = −3.6, *p*_adj_ < 0.01**), but anterior parts did not. In the salience network, SN2 but not SN1 showed increased power (SN2: A-N1: *Z* = −2.1, *p*_adj_ = 0.08, A-N2: *Z* = −3.4, *p*_adj_ < 0.01**). The dorsal attentional DAN, sensorimotor SMN, and both frontoparietal FPN_l,r_ networks also showed no increase in ISF power levels.

Overall, the ISF power levels were higher in RSNs than in NF components. In line with our hypothesis, ISF power increased during sleep, encompassing especially NF components and posterior RSNs.

### Canonical resting-state networks predict signal changes in neurofluid compartments

Although the functional connectivity between the RSNs have been rigorously studied over the years, it remains unknown how the NF components are coupled to the classical RSNs. As the ISF changes in T2*-weighted BOLD fMRI signal mainly reflects relative de-/oxyhemoglobin changes in brain tissue post-capillary veins, it follows that flow changes in the NF, especially venous components, could potentially affect RSN signal changes. To further map the connections between NF components and functional RSNs, we calculated phase TE between ICs in the ISF range (Fig. 2*e*).

During wakefulness, surrogate-normalized directional coupling between RSNs and NF components was predominantly directed from RSNs to NF components (Fig. 2*e*). In addition to RSNs, both CSF and arterial components also predicted changes in venous components. In particular, SN1 and SN2, DMN_a,p_, and Vis_m_ networks predicted activity in the venous sinuses, with component S5 acting as the most prominent receiver. SN2 and DMN_p_ also predicted DAN changes.

During NREM-1 sleep, most RSNs continued to predict venous components. Significant directional influences were observed from SMN and SN2 to venous components S5 and S1 after surrogate testing and FDR correction (*p*_adj_ < 0.05*). In addition, the lateral ventricular component (CSF2) and the arterial component predicted activity in SMN, DAN, and venous components.

During NREM-2 sleep, the SN2 salience network predicted both frontoparietal networks (FPN_l,r_) and SN1, as well as several NF components including the third ventricle–aqueduct system (CSF3) and venous sinus components. As in the awake state, SMN predicted venous components S4 and S5. The medial visual network (Vis_m_) shifted to predict venous sinus component S2. The lateral ventricular component (CSF2) coupled with venous components S3 and S5 and also with the lateral visual network (Vis_l_). The peri-Sylvian CSF component (CSF4), located around the middle cerebral arteries, began predicting neighboring RSNs including SN2, DMN_p_, FPN_r_, and SMN.

Overall, RSNs predominantly predicted NF component dynamics across vigilance states. CSF components also generally predicted venous changes, and during sleep some CSF sources started predicting RSN activity.

To determine which networks were most actively contributing to overall network dynamics, we calculated the degree of coupling, which refers to the number of links to other networks (Fig. 2*f*). We further separated the degree of coupling degree into driving and receiving, thus considering the interaction directionality. Results revealed that SN2, ventricular CSF2, DMN_p_, Vis_m_, SMN, and arterial components were most actively involved in predicting network dynamics, having a net positive effect. In contrast, the venous sinuses S3–5, CSF3, and DAN were especially associated more as receiving nodes, rather than predicting other ICs.

The effective connectivity analysis between networks showed that RSNs predict NF components rather than other way around. The RSN prediction was most coherent during wakefulness, whereas sleep reorganized these functional connections. In line with this, venous sinuses were characterized as receiving nodes, reacting to changes in RSN activity.

### Resting-state networks and neurofluid components exhibit cross-frequency coupling spanning multiple timescales

Using statistically independent signal sources, we analyzed the full detectable frequency spectrum for cross-frequency coupling patterns. We tested the hypothesis that RSNs and NF components exhibit distinct coupling patterns, reflecting their different signal origins.

Raw PAC values revealed three distinct cross-frequency coupling bands within the ISF and cardiorespiratory frequency ranges that were observed consistently between all subjects (Fig. 3*a*). The cardiac amplitudes showed slightly greater variability relative to mean PAC levels, as indicated by the coefficient of variation maps. Surrogate-normalized PAC_n_ maps demonstrated coupling exceeding that expected from intrinsic signal properties alone (Fig. 3*b*). Cluster correction identified significantly deviating clusters from the background, which highlighted cardiorespiratory frequencies as well as portions of the ISF range.

We analyzed three PAC ranges in more detail. The first frequency range exhibited coupling between the ISF phase (∼0.02 Hz) and slow fluctuation (SF) amplitudes (∼0.04–0.11 Hz), which was especially conspicuous during NREM-2 sleep (labeled as “1”). The second range was also most prominent during NREM-2 sleep and involved a wider ISF phase band (∼0.01–0.05 Hz) coupled with respiratory amplitudes (∼0.25 Hz; labeled as “2”). The third coupling range was observed across both wakefulness and sleep and involved respiratory phase (∼0.25 Hz) coupling with cardiac amplitudes (∼1 Hz). Henceforth, we refer to these cross-frequency interactions as follows: 1, ISF–SF; 2, ISF–Resp; and 3, Resp–Card interactions.

With all three coupling patterns, we found that the NF components had clearly higher (*p* < 0.001***) coupling magnitudes in comparison with the RSN (Fig. 3*c*), which was seen robustly across arousal states (Table S2). Moreover, we found that sleep was also associated with increased PAC values in the ISF–SF and ISF–Resp couplings (Fig. 3*c*). With ISF–SF coupling, the median PAC was 0.174 (IQR = 0.172) in awake state, 0.230 (IQR = 0.309) in NREM-1 sleep, and 0.356 (IQR = 0.277) in NREM-2 sleep. The difference in medians between awake and NREM-2 sleep was statistically significant (*Z* = −2.83, *p*_adj_ < 0.05*). Similarly, ISF–Resp coupling during awake state had a median PAC 0.120 (IQR = 0.117), which increased to 0.172 (IQR = 0.265) in NREM-1 sleep, and 0.261 (IQR = 0.221) in NREM-2 sleep, where the difference between awake-NREM-2 was statistically significant (*Z* = −2.83, *p*_adj_ < 0.05*). For full results, see also Table S3.

As expected, we identified statistically significant cross-frequency coupling region that was not due to intrinsic signal properties. This region spanned the cardiorespiratory frequencies and overlapped with the ISF range. The coupling regions studied were more strongly expressed in NF components and had tendency to increase during sleep.

### Infra-slow fluctuation dynamics predict slow fluctuations and respiration

After identifying the frequencies associated with the cross-frequency coupling, a key question remained: in which direction do these interactions take place? To answer this, we further investigated the identified coupling frequency ranges by assessing the phase TE within the interactions (Fig. 4*a*). We hypothesized that the observed PAC coupling patterns would be predicted by the slower signal changes.

To test this hypothesis, we first evaluated the null hypothesis that the distributions of suprathreshold ΔTE would be centered around a zero median, which would suggest no net directionality. Contrary to this hypothesis, we found significant directional information transfer in the ISF–SF (ΔTE_A_ = 0.084 bit, *p*_adj_ < 0.001***; ΔTE_N1_ = 0.078 bit, *p*_adj_ < 0.001***; ΔTE_N2_ = 0.070 bit, *p*_adj_ < 0.001***) and ISF–Resp (ΔTE_A_ = 0.067 bit, *p*_adj_ < 0.01**; ΔTE_N1_ = 0.101 bit, *p*_adj_ < 0.001***; ΔTE_N2_ = 0.076 bit, *p*_adj_ < 0.001***) couplings across all arousal states (Fig. 4*b*). In both the ISF–SF and ISF–Resp coupling frequency ranges, the ISF phase acted as the net predictor of faster frequencies. In contrast, for the Resp–Card coupling, a significant net directionality was observed only during NREM-2 sleep (ΔTE*_A_* = −0.0004 bit, *p*_adj_ = 1.0; ΔTE_N1_ = −0.012 bit, *p*_adj_ = 1.0; ΔTE_N2_ = −0.072 bit, *p*_adj_ < 0.01**).

Arousal state had little effect on the ΔTE magnitude; the ISF–SF and ISF–Resp couplings did not show notable changes across wakefulness and sleep stages. However, the Resp–Card interaction showed no net directionality during wakefulness and NREM-1 sleep but shifted to negative ΔTE during NREM-2 sleep, indicating cardiac prediction over the respiratory activity.

All ICs demonstrated consistent prediction directions in the ISF–SF and ISF–Resp couplings. Only small differences were observed between RSN and NF components, suggesting equal expression of coupling in the studied ICs. In contrast, the Resp–Card coupling displayed larger differences between components. While most components displayed cardiac amplitude prediction of respiratory phase, DMN_p_, Vis_m_, and DAN showed respiratory prediction over cardiac changes.

Overall, our results generally support a slow to fast hierarchical organization of the human brain pulsations, as evidenced by the observed directional net prediction patterns. However, contrary to our expectations, cardiac amplitude fluctuations during NREM-2 sleep predicted respiratory changes.

## Discussion

In this study, we examined the coupling of three major physiological brain pulsations, vascular ISF, respiratory, and cardiovascular fluctuations, using a 10 Hz MREG fMRI sequence. These brain-wide pulsations were linked through cross-frequency coupling at ISF–SF, ISF–Resp, and Card–Resp frequency bands. The slower fluctuations predicted faster amplitude changes, with coupling strength progressively increasing from wakefulness to NREM-1 and NREM-2 sleep. Functional brain RSNs predominantly predicted the downstream venous NF compartments, especially during awake state. Collectively, these findings indicate that brain hemodynamics follow hierarchical dynamics.

### Hierarchical organization of human brain pulsations

ISFs are a prominent feature of resting-state brain activity and are observed across multiple systems, including electrophysiological signals, vascular dynamics, and within CSF spaces (Biswal et al., 1995; Kiviniemi et al., 2000; Vanhatalo et al., 2004; Fultz et al., 2019; Väyrynen et al., 2023, 2026). ISFs of electrical activity modulate the power of neural rhythms (Väyrynen et al., 2023), which also together with water volume changes predict changes in cerebral blood flow, through neurovascular coupling mechanisms in awake state (Väyrynen et al., 2026). During NREM sleep, several studies have reported increased power of hemodynamic ISFs (Fultz et al., 2019; Helakari et al., 2022, 2023; Picchioni et al., 2022), which also coincide with altered neurovascular and hydrodynamic coupling (Väyrynen et al., 2026).

Animal studies have suggested that slow vasomotor waves are related to neuromodulatory processes, namely, fluctuating norepinephrine levels, originating from the locus ceruleus (Hauglund et al., 2025), while larger mammal noninvasive studies show BOLD anticorrelation in nucleus basalis of Meynert (Liu et al., 2018).

Our results here showed that hemodynamic ISFs are coupled to slow fluctuations (ISF–SF) as well as respiratory amplitudes (ISF–Resp). The respiratory changes were in turn coupled with (Resp–Card) cardiac amplitudes. Predictive analysis further revealed information transfer predominantly from slow to faster rhythms, suggesting that the ISF phase may act as a coordinator of higher-frequency pulsations. Similar hierarchical slow-to-fast organization occurs with many physiological systems, for example, circadian rhythms coordinating hormone release (Gnocchi and Bruscalupi, 2017), the respiratory cycle influencing heart rate in respiratory sinus arrhythmia (Yasuma and Hayano, 2004), and slow electric fluctuations in brain modulating fast cortical processing (Väyrynen et al., 2023).

Although our results largely support a slow-to-fast model of hierarchical organization of brain pulsations, cardiorespiratory coupling deviated from this general rule. Others have established that cardiorespiratory coupling can have bidirectional dynamics, with various physiological regulation mechanisms within the thorax (Barnett et al., 2021). Here, awake and NREM-1 states TE demonstrated no net interaction direction in either way, but cardiac pulsatility during NREM-2 sleep started predicting respiratory fluctuations. This suggests the occurrence of altered cardiorespiratory coupling during deeper sleep, with reversed fast-to-slow modulation of cardiorespiratory coupling.

### Component-level insights into neurovascular coupling

The BOLD contrast in the low-frequency range reflects a mixture of signal sources that vary by spatial location. In this frequency domain, functionally connected standing waves of neural activity and propagating vasomotor waves over the brain cortex contribute ISF BOLD signal variance (Bolt et al., 2022). These two mechanisms share overlapping frequencies, making them difficult to disentangle. However, the spatial ICA detects only stationary signal sources emphasizing the neuronal RSN effects more on the coupling patterns in relation to NF domains. The PAC coupling strength was higher with NF components, and it increased during sleep. Even though the PAC and ISF power increased in sleep (Figs. 2, 3), the TE strength of prediction was not altered during sleep (Fig. 4*b*).

TE analysis further indicated that ISF RSN activity almost exclusively predicted NF component dynamics during wakefulness, where also CSF compartments predicted venous sinus changes (Fig. 2*e*).This result is consistent with classical activation hyperemia, whereby cortical RSN activity increases the local cerebral blood flow, which is reflected successively into CSF and venous sinuses sources due to volume displacement as indicated by the Monro–Kellie doctrine (Fultz et al., 2019; Hauglund et al., 2025; Väyrynen et al., 2026). During sleep, these relationships became more complex and were no longer predicted as exclusively top-down cortical sources; CSF sources during sleep increased their prediction toward RSNs, which could be related to increase in ISF power. We suppose that the increased pulsation power in CSF could induce up-stream effects along structures in the RSNs due to altered coupling electro-, hydro-, and hemodynamics in sleep (Väyrynen et al., 2026).

The ICA dimension reduction and following surrogate approach helped us to assess whether observed cross-frequency coupling patterns were genuine or arising by chance. Indeed, significant coupling frequency ranges assessed at the component level differed from voxel level results. For example, an apparent ISF–Card coupling observed at the voxel level may have spuriously resulted from high autocorrelation of the propagating arterial impulses, rather than representing a genuine coupling, as also supported by slightly elevated intersubject variance (Fig. 3). In contrast, surrogate statistics also revealed a cardiorespiratory coupling pattern that was not evident at the voxel level. Notably, this proved to be the most stable coupling pattern across all arousal states, while ISF-related couplings were more apparent during NREM-2 sleep. PAC magnitudes depend on signal powers, and sleep increases ISF power, suggesting that the elevated PAC levels observed during sleep (Figs. 1, 3) could also relate to an increased power level. Future studies should explore complementary control strategies in addition to those introduced here, for example, time-reversal tests, to further validate the directional coupling estimates obtained here.

### Sleep-dependent amplification of infra-slow dynamics

ISF power was consistently higher in the RSNs than in the NF sources, most likely due to oxygenation level shifts occurring more prominently at the brain tissue level than in blood or CSF. The sleep-associated increase in ISF power across both RSN and NF components aligns with prior evidence in mice and humans (Iliff et al., 2012; Fultz et al., 2019; van Veluw et al., 2020; Helakari et al., 2022; Hauglund et al., 2025; Väyrynen et al., 2026). The increase in relative ISF power with respect to total signal power (Fig. 2*c*) suggests that this was not due to elevation in overall signal power levels but rather reflects true increase within the ISF band, an interpretation also supported by the absolute power results (Fig. 2*b*). Notably, some ICs exhibited low relative ISF power levels, largely due to high cardiorespiratory pulsatility, which significantly reduced ISF power relative to total signal power levels.

In mice, decreased locus ceruleus activity, and associated ∼0.02–0.03 Hz fluctuations in cortical norepinephrine levels during sleep, entrains vasomotor fluctuations in vascular walls, enhancing perivascular CSF flow (Osorio-Forero et al., 2021; Kjaerby et al., 2022; Hauglund et al., 2025). We have previously shown that MREG ISF amplitudes (Helakari et al., 2022; Väyrynen et al., 2026) and brain interstitial fluid and CSF flow speeds (Elabasy et al., 2026) increase locally during sleep. We have further shown sleep-related alterations in neurovascular and hydrodynamic coupling (Väyrynen et al., 2026).

Our present findings extend this model; declining norepinephrine levels during NREM-1 and NREM-2 sleep permit increased perturbations within arterial walls, which manifests as increased ISF power in arterial IC components but also downstream (peri)venous components, which dominate the increases in the BOLD signal. This pattern may reflect reduced orexinergic drive (Järvelä et al., 2025), resulting in decreased tonic locus ceruleus activity, leading to increased fluctuations in the norepinephrine levels and also enhanced vascular wall fluctuations (Hauglund et al., 2025).

Changes in NE related infra-slow pupillary changes have been linked to the occurrence of sleep spindles and microarousals in humans (Carro-Domínguez et al., 2025). Interestingly, the phase of EEG ISFs correlates with the occurrence of arousal state change related K-complexes (Vanhatalo et al., 2004) and predicts neural amplitudes (Väyrynen et al., 2023). EEG slow-wave activity has been suggested to induce CSF inflow in humans (Fultz et al., 2019) and anesthetized mice (Hablitz et al., 2019). More recently, glymphatic influx and clearance were found to correlate more strongly with infra-slow NE fluctuations and the occurrence of microarousals (Hauglund et al., 2025).

Multiple EEG sleep features appear to be closely related to infra-slow changes and glymphatic function, highlighting the need for future studies to further characterize these interactions, particularly in humans.

### Functional implications for neurofluid circulation

Beyond modulatory PAC effects, we hypothesize that transient phase alignment among ISF, respiratory, and cardiac pulsations could create constructive interference and thereby amplify pressure gradients to promote CSF transport. Conversely, desynchronization induced by changes in vascular stiffness or brain activity state could result in destructive interference, which could diminish CSF bulk flow. This conceptual framework could provide a mechanistic link between sleep, pulsation synchrony, and CSF flow, with implications for pathologies such as Alzheimer's disease, which are characterized by altered brain pulsatility.

Together, these findings suggest that human brain pulsations form a tightly interconnected hierarchical system, in which slow hemodynamic changes may coordinate faster pulsatory events. The observed strengthening of coupling patterns during NREM sleep supports the notion of increased CSF drive, in association with enhanced brain solute transport during sleep.
